# CKAP4 and PLOD2 as novel prognostic biomarkers in hepatocellular carcinoma: a proteomics-driven risk stratification model

**DOI:** 10.3389/fcell.2025.1577161

**Published:** 2025-07-02

**Authors:** Qiulong Lu, Zhao Cao, Yueting Xiong, Junqiang Huang, Huan Zeng, Zhijian Chen, You Shu, Yahan Tan, Xiaoling Long, Xiaohui Liu, Hong Shu

**Affiliations:** 1 Department of Clinical Laboratory, Guangxi Medical University Cancer Hospital, Nanning, China; 2 Department of Clinical Laboratory, First Affiliated Hospital of Guangxi Medical University, Nanning, China; 3 State Key Laboratory of Vaccines for Infectious Diseases, Xiang An Biomedicine Laboratory, Xiamen University, Xiamen, China; 4 Institute of Translational Medicine, Shanghai Jiao Tong University, Shanghai, China

**Keywords:** HCC, DIA-MS proteomics, prognosis biomarkers, prognostic model, PLOD2, CKAP4

## Abstract

**Background:**

The prognosis of patients with hepatocellular carcinoma (HCC) is a research hotspot. This study aimed to identify novel prognostic protein markers for HCC using data-independent acquisition mass spectrometry (DIA-MS) and develop an integrative predictive model to enhance clinical decision-making and patient stratification.

**Methods:**

DIA-MS were implemented to identify valuable prognostic HCC biomarkers in 31 patients with different prognoses. A prognostic model was developed and validated using immunohistochemistry (IHC).

**Results:**

Cytoskeleton-associated membrane protein 4 (CKAP4) and procollagen-lysine, 2-oxoglutarate 5-dioxygenase 2 (PLOD2) were identified as key prognostic proteins, with higher expression levels associated with poor prognosis. Immunohistochemical validation confirmed the prognostic value of CKAP4 and PLOD2. A nomogram incorporating AJCC stage and the combination of CKAP4 and PLOD2 demonstrated superior predictive Sability for overall survival (OS) compared to individual indicators. The model predicted an outcome with a concordance index (C-index) of 0.738 (95% CI, 0.698–0.779) and significantly stratified patients into distinct risk groups (*P* < 0.001).

**Conclusion:**

In conclusion, this study identified CKAP4 and PLOD2 as novel prognostic protein markers for HCC. The developed nomogram, integrating these molecular markers with AJCC stage, shows promise in predicting OS and stratifying risk in HCC patients.

## Graphical Abstract

**Figure:**
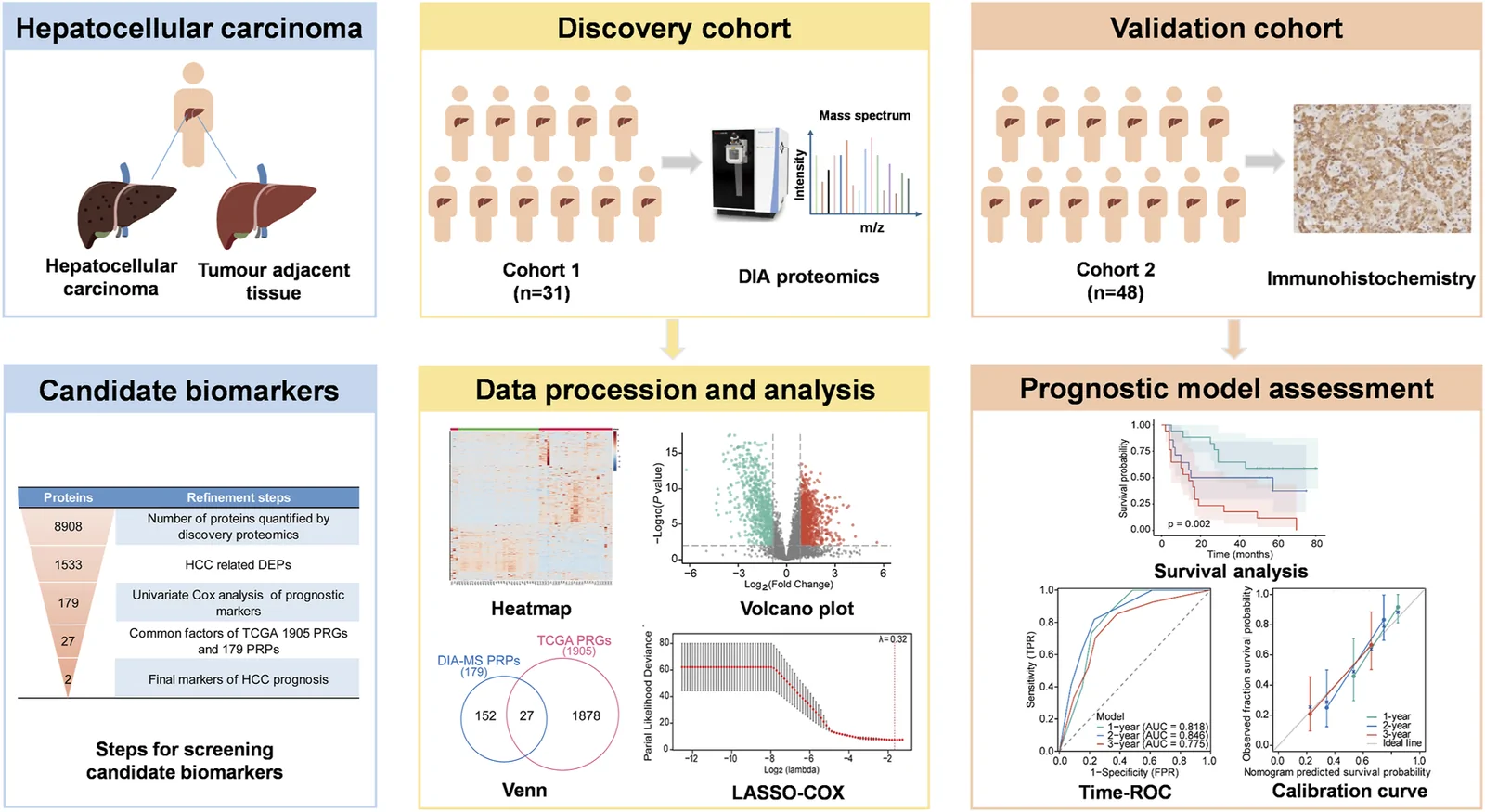
Schematic overview of the study design. DEPs, differentially expressed proteins; PRGs: prognosis-related genes; PRPs, prognosis-related proteins.

## 1 Introduction

Hepatocellular carcinoma (HCC) persists as a formidable global health challenge, ranking as the sixth most prevalent malignancy and the third leading cause of cancer-related mortality worldwide. The annual incidence surpasses 905,000 new cases, resulting in over 830,000 deaths (Bray et al., 2018; Forner et al., 2018). Despite significant advancements in therapeutic modalities, the prognosis for HCC patients remains dismal, primarily due to the high propensity for recurrence and metastasis, exacerbated by the frequent presence of underlying liver dysfunction (Tateishi et al., 2005).

Serum biomarkers alpha-fetoprotein (AFP) and vitamin K absence/antagonist-II (PIVKA-II) play complementary roles in HCC prognosis. AFP reflects hepatocytic differentiation and associates with vascular invasion and early recurrence while PIVKA-II, indicative of angiogenic activity, shows superior sensitivity for microvascular invasion (Park et al., 2014; Hynes et al., 2024). Their combined use in models like the GALAD score enhances recurrence risk stratification (Cagnin et al., 2023). However, circulating biomarkers lack spatial resolution to assess intratumoral heterogeneity.

Surgical resection remains the gold standard for curative treatment in early-stage HCC. However, its efficacy is significantly compromised by the heterogeneity in post-resection outcomes. Notably, the postoperative recurrence rate remains alarmingly high, ranging from 60% to 70% (Chan et al., 2015; Dhir et al., 2016). While serum biomarkers provide valuable prognostic information, they lack spatial resolution to assess intratumoral molecular heterogeneity, a critical determinant of recurrence patterns. This high recurrence rate has emerged as the primary determinant of poor prognosis in post-surgical HCC patients. Consequently, there is an urgent need for integrated prognostic models that combine circulating biomarkers with tissue-specific molecular markers to accurately stratify patients based on their recurrence risk, guide the development of personalized treatment strategies, and inform more effective post-operative surveillance protocols.

Current prognostic tools, including the Child-Pugh scoring system, Cancer of the Liver Italian Program (CLIP), and Barcelona Clinic Liver Cancer (BCLC) staging systems, exhibit limitations in their applicability across diverse patient subgroups (Trevisani et al., 2024; Johnson et al., 2022; Kudo et al., 2003). While numerous potential biomarkers have been identified in blood and tumour tissues, many studies focus on single genes or proteins, which are insufficient for comprehensive HCC prognostication (Gao et al., 2019; Zhang et al., 2021; Che et al., 2021). Moreover, the clinical validation of these biomarkers remains limited, underscoring the need for more integrative approaches.

Recent advancements in mass spectrometry technologies, characterized by enhanced sensitivity, resolution, and accuracy, coupled with sophisticated bioinformatics tools, have revolutionized proteomic research (Zhang et al., 2024). The data-independent acquisition (DIA) mass spectrometry mode, in particular, offers superior proteome coverage, reproducibility, and quantification accuracy (Kitata et al., 2022). This technological progress has facilitated the identification of novel DEPs as potential prognostic biomarkers in cancer research.

In this study, we employed DIA-MS quantitative proteomic analysis on 31 paired HCC and adjacent non-tumour tissue samples to identify prognostic protein markers. We developed a prognostic model using Least Absolute Shrinkage and Selector Operation (LASSO) -COX regression and evaluated the predictive value of key proteins through survival analysis. Furthermore, we integrated these molecular markers with clinical features to construct a nomogram for HCC prognosis prediction. Our approach aims to enhance the accuracy of HCC progression prediction and identification of high-risk patients, potentially improving clinical decision-making and patient outcomes.

## 2 Materials and methods

### 2.1 Clinical sample collection

This study comprised two independent cohorts of HCC patients. Cohort 1 included paired HCC and adjacent non-tumour tissue samples from 31 patients who underwent hepatectomy for liver surgery at the specimen repository of Guangxi Medical University Cancer Hospital, with samples collected between 2016 and 2018 (Supplementary Table S1). The distinction between HCC tissues and adjacent non-tumour tissues is based on intraoperative rapid frozen pathological assessment. Subsequently, the diagnosis was confirmed by two independent pathologists to ensure the accuracy and reliability of the assessment. The 31 paired samples were used for proteomic characterization, and the follow-up data for these patients had a cutoff date of May 2021. Additionally, Cohort 2 consisted of 48 patients with HCC undergoing radical resection at Guangxi Medical University First Affiliated Hospital between 2017 and 2019 (Supplementary Table S2). This cohort was utilized for immunohistochemical validation, and the follow-up data had a cutoff date in September 2023. This study was approved by the Research Ethics Committee of the Guangxi Medical University Cancer Hospital (2019 (KY-E-18), LW2024130) and the First Affiliated Hospital of Guangxi Medical University [2019 (KY-E-086)].

### 2.2 Sample preparation

Tissue samples were homogenized in lysis buffer (abs9229, Absin, China) and subjected to sonication. The homogenates were centrifuged at 13,000 × g for 20 min at 4°C. Protein and peptide concentrations were determined using the BCA assay (23,225, Thermo Scientific, United States). The supernatants were collected and stored at −80°C for further analysis. Proteins were extracted using a lysis buffer containing 1% sodium deoxycholate (DOC), 10 mM tris (2-carboxyethyl) phosphine (TCEP), 40 mM chloroacetamide (CAA), and 100 mM Tris-HCl (pH 8.5). ProteaseMAX surfactant solution (Promega Corporation, United States) was added to a final concentration of 0.01%. The mixture was heated at 95°C for 5 min and then subjected to ultrafiltration. The protein digest was diluted with 100 mM tetraethylammonium bromide (TEAB) to a final concentration of 1 μg/μL. Lysyl endopeptidase (Lys-C) and trypsin were added at appropriate ratios, along with ProteaseMAX surfactant solution to a final concentration of 0.02%. The digestion was carried out at 37°C for 30 min and quenched with 10% trifluoroacetic acid (TFA). Finally, the peptides were desalted using a C18 solid-phase extraction column (2 μm, 75 μm × 500 mm, WAT054960, Thermo Scientific, United States) and then lyophilized. For the LC-MS analysis, the peptide concentration was standardized to 1 μg per sample.

### 2.3 DIA-based LC-MS/MS

Peptide analysis was performed using an Orbitrap Exploris 480 mass spectrometer equipped with field asymmetric ion mobility spectrometry (FAIMS) coupled to an EASY-NanoLC 1,200 system (both from Thermo Fisher Scientific, MA, United States). Peptides were reconstituted in 0.1% formic acid (FA) prior to analysis. Chromatographic separation was achieved using an Acclaim PepMap C18 analytical column (75 μm × 25 cm) with a 130-min gradient. Data-independent acquisition (DIA) mode was employed for LC-MS/MS analysis of peptides from each sample. FAIMS compensation voltages (CV) were set at −45 and −65. MS scans were acquired over an m/z range of 350–1,200 with a resolution of 120,000. The automatic gain control (AGC) target was set to 300 with a custom maximum injection time. HCD-MS/MS was performed at a resolution of 30,000, with an AGC target of 200 and collision energies of 25, 30, and 35. The column flow rate was maintained at 250 nL/min. Quality control samples, consisting of pooled aliquots from all experimental samples, were analyzed in DIA mode at the beginning of the MS study and after every six injections to monitor instrument performance.

### 2.4 MS spectrometry data analysis

Raw data processing and analysis were performed using Spectronaut 14 (Biognosys AG, Switzerland). Database searches for spectral library generation were conducted against the UniProt human database (20,416 entries). Search parameters included a maximum of two missed cleavages, a minimum peptide length of six amino acids, and a maximum of three modifications per peptide. The Q-value (false discovery rate, FDR) thresholds for both library generation and DIA analyses were set to 0.01. The MS proteomics data have been deposited to the ProteomeXchange Consortium (http://proteomecentral.proteomexchange.org) via the iProX partner repository with the dataset identifier PXD051768 (Ma et al., 2019; Chen et al., 2021).

### 2.5 DEPs identification and functional analysis

Protein expression in HCC tissues was compared to adjacent non-tumour liver tissues. Proteins detected in <50% of samples were excluded, and missing values were imputed with half the minimal value for each protein. DEPs were defined as those with a fold change (FC) > 1.80 or <0.56 and a paired t-test *P*-value <0.01. Partial least squares discriminant analysis (PLS-DA) was employed to assess the relationship between protein expression and sample types.

Protein expression values were normalized to address technical variability and batch effects. The workflow comprised: (1) Total Protein Normalization (TPN) to adjust for inter-sample variations in total protein content; (2) Log10 transformation to stabilize variance across the dynamic range of protein abundances; (3) Pareto scaling to mitigate dominance by high-abundance proteins. To generate the hierarchical clustering heatmaps, the data was first standardized by autoscaling to normalize the dataset. The Pearson correlation coefficient was used as the distance measure, and the average linkage method was applied for clustering. A heatmap with hierarchical clustering analysis was generated based on normalized protein values to provide an overview of DEP characteristics. Gene Ontology (GO) analysis was conducted using the STRING database (https://string-db.org/), focusing on biological processes, molecular functions, and cellular components. The Kyoto Encyclopedia of Genes and Genomes (KEGG) enrichment analyses were conducted utilizing the Metascape database (https://metascape.org/) (Zhou et al., 2019), with the screening parameters established as a minimum overlap of 3 and a minimum enrichment factor of 1.5.

### 2.6 Construction and validation of a prognostic model

To investigate the relationship between protein expression levels and overall survival (OS) of HCC patients, univariate Cox regression analyses were performed. Proteins with *P* < 0.05 were selected for further analysis. To eliminate gene collinearity, LASSO-COX regression analysis was conducted using the R package “glmnet”. The optimal penalty parameter λ was determined by 3-fold cross-validation. Risk scores were calculated as the sum of each protein’s coefficient multiplied by its expression level. Patients were classified into low- and high-risk groups based on the median risk score. Kaplan-Meier curves were generated using the R package “survminer” to compare OS between groups using a log-rank test. The model refinement process is summarized in the graphical abstract and Supplementary Material.

The predictive ability of the signature was validated using an independent cohort. Univariate and multivariate Cox regression analyses were performed to determine whether the combination of CKAP4 and PLOD2 was an independent prognostic factor for OS in HCC patients. Nomograms were generated from Cox regression coefficients using the “rms” package. Time-dependent receiver operating characteristic (ROC) curves were used to evaluate the predictive ability of CKAP4, PLOD2, and related clinical parameters for 1-, 2-, and 3-year OS in the validation set. X-tile (version 3.6.1) was used to determine suitable cut-off points for the risk score (Camp et al., 2004). The clinical utility of the model was assessed using the C-index, decision curve analysis (DCA), calibration curves, and time-dependent ROC curves.

### 2.7 Immunohistochemistry

Liver samples from HCC patients were fixed, paraffin-embedded, and sectioned using standard methods. Sections were deparaffinized, hydrated, and subjected to antigen retrieval. Nonspecific binding was blocked with sheep serum albumin. Sections were incubated with primary antibodies against CKAP4 (16,686-1, Proteintech, China) and PLOD2 (66,342-1, Proteintech, China). Secondary antibody incubation was carried out using a universal two-step assay kit (PV9000, Zsbio, China), followed by staining with diaminobenzidine (AR1027, Boster, China). After counterstaining with hematoxylin (C0107, Beyotime, China), the sections were dehydrated and mounted. Image analysis was performed using Image-Pro Plus 6.0.

### 2.8 Statistical analysis

Statistical analyses were conducted using SPSS 22.0 and R software (version 3.6.3). Data are expressed as mean ± standard deviation (SD). Statistical significance between two groups was determined using Student’s t-test. A two-tailed *P*-value < 0.05 was considered statistically significant.

## 3 Results

### 3.1 Global proteome characterization of HCC and adjacent non-tumour liver tissues

**FIGURE 1:**
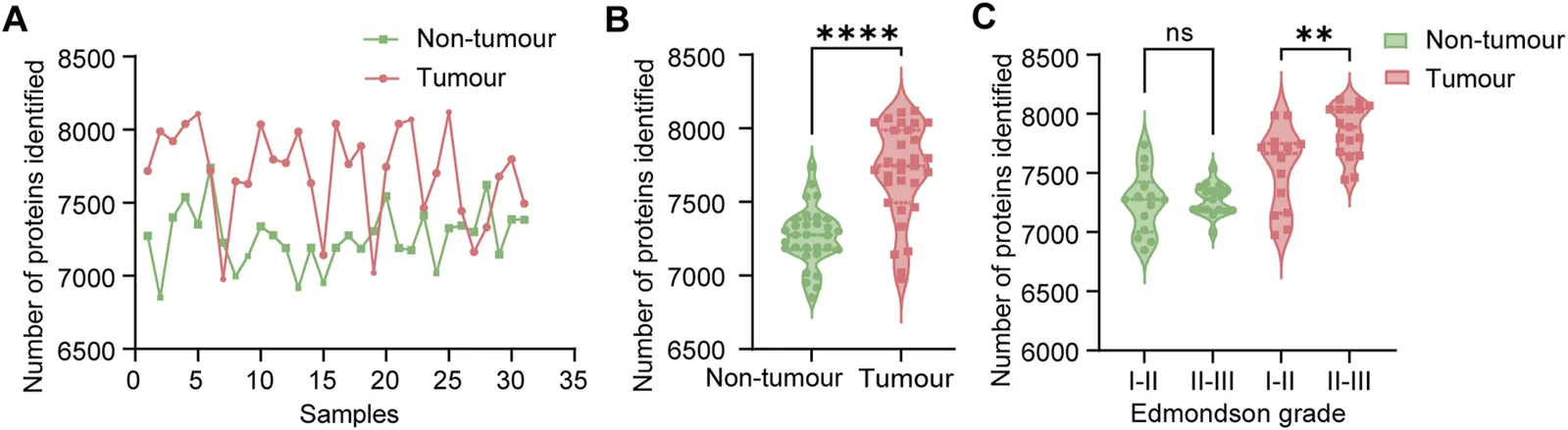
Comprehensive proteomic analysis of hepatocellular carcinoma (HCC) patient samples. **(A)** Protein identification in paired liver tissue samples (n = 31 pairs). Pink bars represent normal liver tissue; green bars represent HCC tumour tissue. **(B)** Violin plots depicting the distribution of identified proteins in normal (pink, n = 31) and tumour (green, n = 31) liver tissues. Statistical significance was determined using a paired t-test (****P < 0.0001). **(C)** Comparison of protein identification in tumour tissues stratified by Edmondson grade. Statistical analysis was performed using one-way ANOVA with Tukey’s *post hoc* test (ns: not significant, *P* > 0.05; **P < 0.01).

DIA quantitative proteomics was conducted on 31 paired cancerous and non-tumour samples from HCC patients, identifying 8,908 unique proteins. The average number of proteins identified in HCC liver tissue was 7,705, compared to 7,264 in normal liver tissue. The mean number of proteins identified in HCC liver tissue (7,705) significantly exceeded that in normal liver tissue (7,264) (Figure 1A,B). Moreover, patients with high Edmondson grades exhibited a greater number of identified proteins in HCC tissue (Figure 1C). These results show the differences in protein expression quantities between HCC tissue and normal liver tissue, as well as the relationship between protein numbers in HCC tissue and Edmondson grades.

**FIGURE 2:**
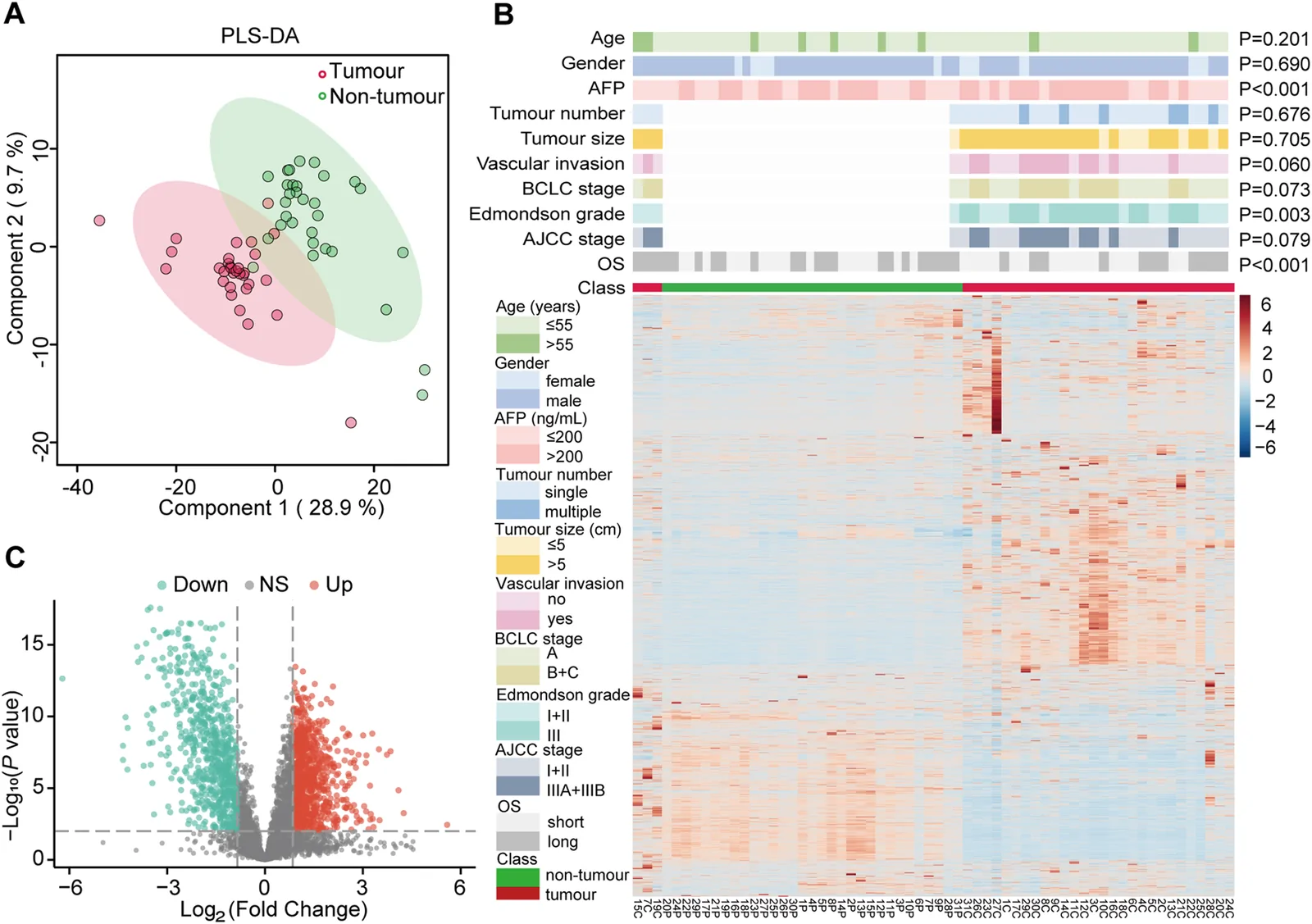
Identification of differentially expressed proteins (DEPs) and their correlation with clinicopathological features of hepatocellular carcinoma (HCC). **(A)** Partial least squares discriminant analysis (PLS-DA) of protein expression profiles in HCC tumours (pink, n = 31) and paired adjacent normal tissues (green, n = 31). **(B)** Hierarchical clustering heatmap of DEPs in HCC tumours **(C)** and paired adjacent normal tissues (P). Sample annotations are indicated by colored bars below the heatmap, representing tumour status (red: tumour; green: non-tumour), age, gender, alpha-fetoprotein (AFP) levels, tumour number, tumour size, vascular invasion status, Barcelona Clinic Liver Cancer (BCLC) stage, Edmondson grade, TNM stage, overall survival (OS), and tumour classification. **(C)** Volcano plot illustrating the distribution of DEPs between HCC and adjacent normal tissues. Red dots represent significantly upregulated proteins, green dots represent significantly downregulated proteins, and gray dots (NS) indicate proteins with no significant differential expression.

Quality control sample correlation analysis demonstrated significant concordance, with heat map analysis revealing strong correlation coefficients between HCC and non-tumour groups (Supplementary Figure S1A,B). High inter-experiment correlation coefficients evidenced robust stability and reproducibility. Partial least squares discriminant analysis (PLS-DA) further confirmed this distinct pattern (Figure 2A). The heatmap clearly delineated protein-level differences between tumour and adjacent non-cancerous tissues, supporting subsequent comparisons (Figure 2B).

### 3.2 Proteomic features of DEPs in HCC

To assess the significance of differential protein expression, we performed t-tests on the FC of each protein in the HCC group compared to the adjacent non-tumour group. DEPs were defined as those with *P* < 0.01 and FC > 1.80 (up-regulated) or FC < 0.56 (down-regulated). This analysis yielded 1,533 DEPs (Supplementary Table S3), comprising 866 up-regulated and 667 down-regulated proteins in the HCC group. A volcano plot visualized the statistical distribution of DEPs in HCC compared to adjacent non-tumour tissue (Figure 2C).

To elucidate the potential functions of DEPs, we conducted functional enrichment analysis. Gene Ontology (GO) analysis encompassed biological processes, cellular components, and molecular function annotations, providing a comprehensive functional overview of the DEPs. Proteins upregulated in hepatocellular carcinoma (HCC) patients compared to adjacent non-tumour tissues were significantly enriched in small molecule catabolism, amino acid metabolism, fatty acid metabolism, and organic acid catabolism, among other biological processes. Cellular component annotation revealed that the majority of DEPs were localized to the mitochondrial matrix, peroxisomes, and organelle inner membranes. Molecular function analysis demonstrated an enrichment in oxidoreductase activity (Supplementary Figure S2A). Additionally, we performed Kyoto Encyclopedia of Genes and Genomes (KEGG) pathway-based enrichment analysis of the DEPs. Results indicated significant involvement in energy metabolism, carbohydrate metabolism, signaling pathways, post-translational protein modifications, and protein transport (Supplementary Figure S2B).

### 3.3 Establishment and validation of a prognostic model based on CKAP4 and PLOD2 in hepatocellular carcinoma

**FIGURE 3:**
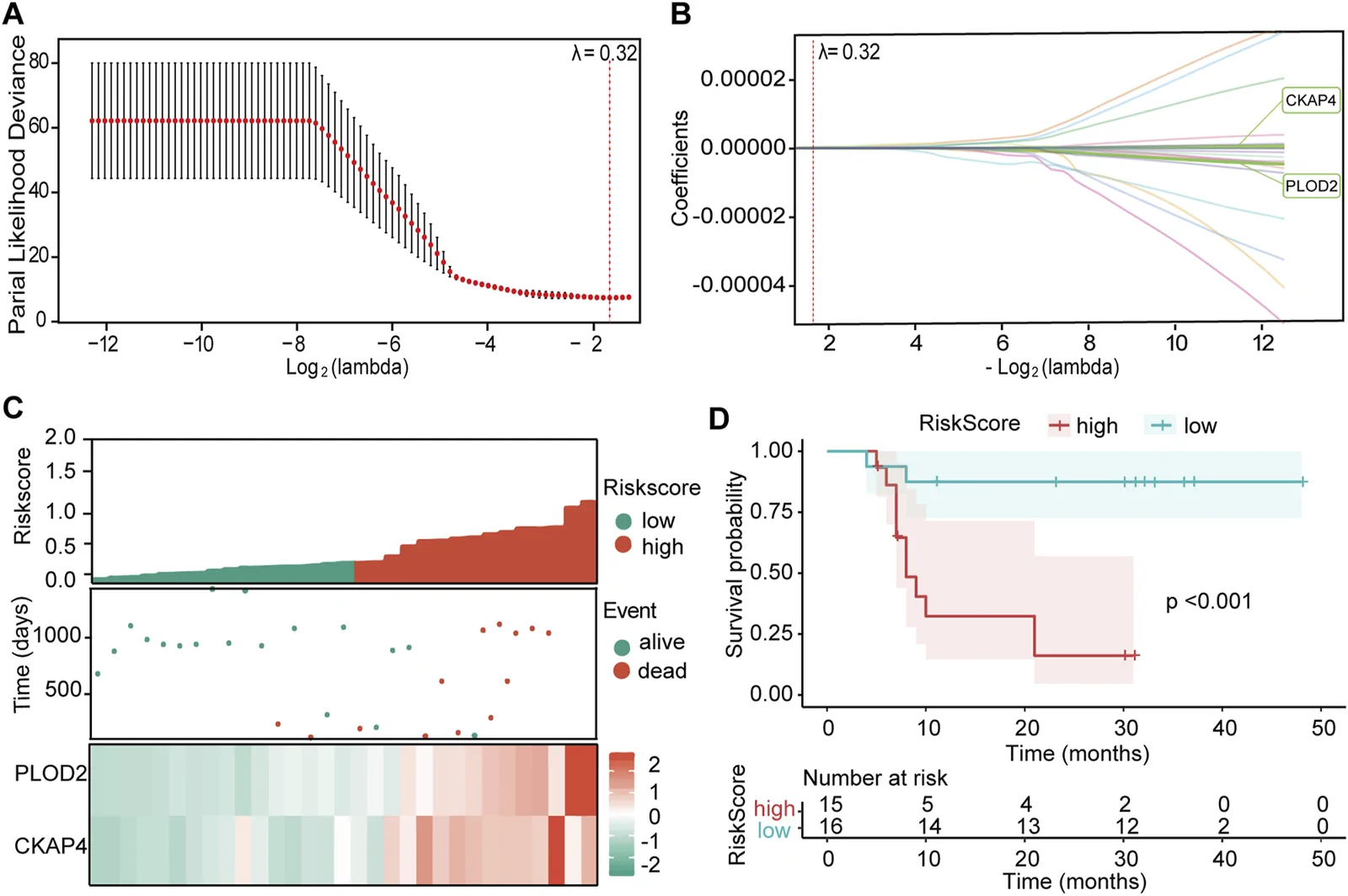
Development and validation of the prognostic model. **(A)** LASSO regression analysis of 27 overall survival (OS)-related proteins. Cross-validation plot for the LASSO-Cox regression model to determine the optimal tuning parameter (λ). The x-axis represents log(λ) values, while the y-axis shows partial likelihood deviance. The λ value yielding the minimum mean 3-fold cross-validated error was selected. **(B)** LASSO-Cox regression coefficient profile plot for variable selection. The vertical dashed line indicates the optimal λ value that minimizes the mean cross-validated error. **(C)** Distribution of risk scores, patient survival status, and heatmap depicting the expression levels of CKAP4 and PLOD2 in the study cohort. **(D)** Kaplan-Meier survival analysis stratified by risk score for predicting overall survival. The *p*-value was calculated using the log-rank test.

From 1,533 DEPs, univariate Cox regression analysis identified 179 prognosis-related proteins (*P* < 0.05, Supplementary Table S4). Comparison of prognosis-related proteins (PRPs) from our cohort with prognosis-related genes from The Cancer Genome Atlas (TCGA)-LIHC cohort (Supplementary Table S5) revealed 27 common signatures (Supplementary Table S6). These proteins underwent a multistage refinement process summarized in graphical abstract. Subsequent LASSO-Cox regression analysis (Figure 3A,B) resulted in a prognostic model incorporating CKAP4 and PLOD2. The prognostic index was calculated as:

$$\begin{array}{ll} Risk\;score & =\left(CKAP4\;expression\;*\;\mathrm{1.07370337029954}e-\mathrm{08}\right)\\ & \;+\left(PLOD2\;expression\;*\;\mathrm{7.98110767056107}e-\mathrm{08}\right) \end{array}$$

To validate the model’s predictive capacity, patients were stratified into high-risk (N = 15) and low-risk (N = 16) groups based on the median risk score (Figure 3C). The high-risk group exhibited higher mortality rates and shorter survival times, with overexpression of both CKAP4 and PLOD2. Kaplan-Meier analysis confirmed poorer prognosis in the high-risk group (*P* < 0.001, Figure 3D).

ROC curve analysis demonstrated the model’s stability (Supplementary Figures S3A,B). In the discovery cohort, the model showed a sensitivity of 0.944 and specificity of 0.769, with an area under the curve (AUC) of 0.915. Time-dependent ROC analysis yielded AUCs of 0.899, 0.951, and 0.962 for 1-, 2-, and 3-year OS, respectively. A positive correlation was observed between CKAP4 and PLOD2 expression levels (R = 0.72, *P* < 0.001, Supplementary Figure S4A). These results underscore the model’s robust performance in predicting HCC prognosis.

### 3.4 Validation of prognostic proteins by immunohistochemical analysis

**FIGURE 4:**
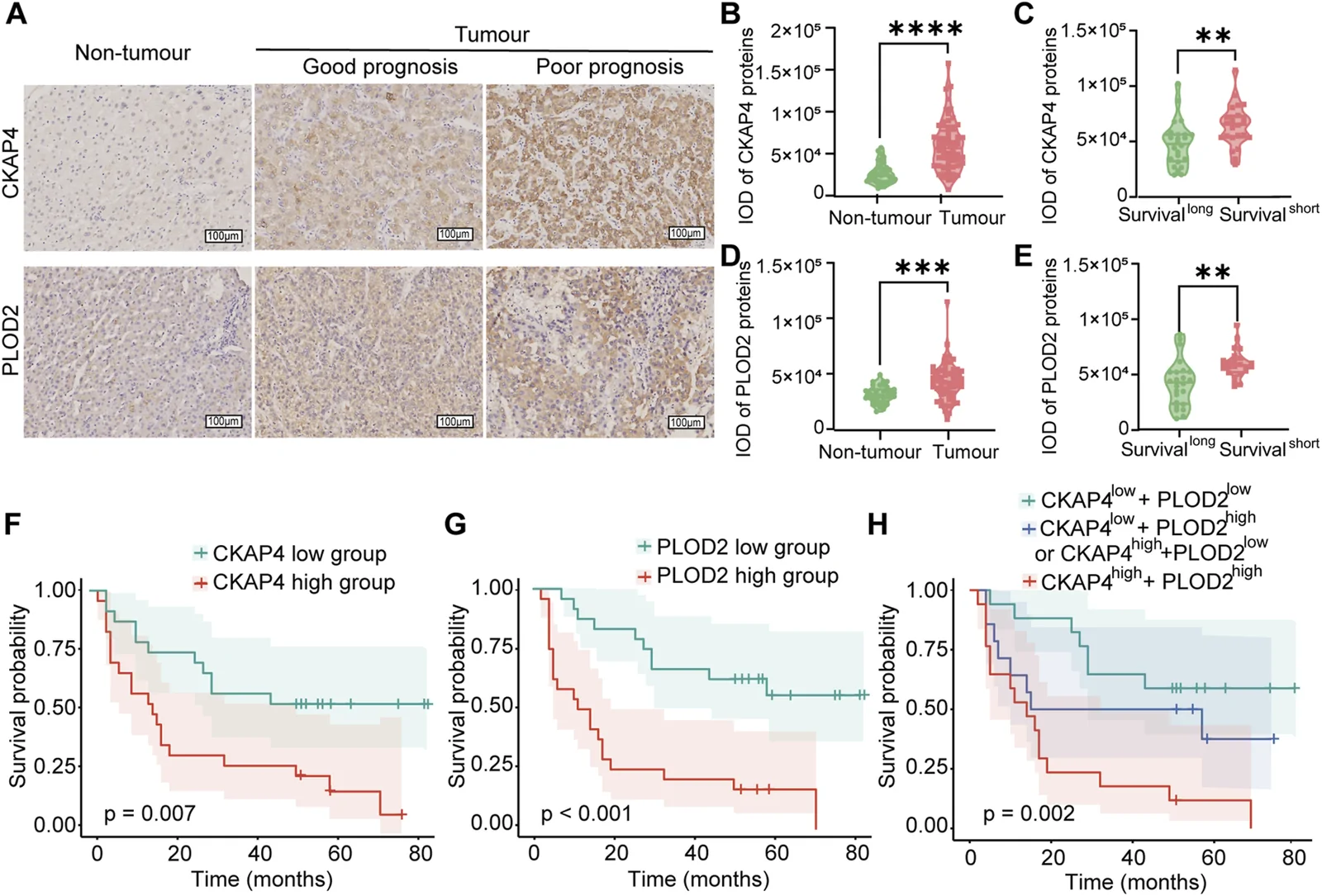
Validation of CKAP4 and PLOD2 expression in relation to HCC progression. **(A)** Representative immunohistochemistry (IHC) images illustrating CKAP4 and PLOD2 expression in hepatocellular carcinoma (HCC) tissues (n = 48) and adjacent non-tumour tissues (n = 38). Scale bar: 100 μm. **(B–E)** Quantification of CKAP4 and PLOD2 expression using integrated optical density (IOD) values in HCC and adjacent non-tumour liver tissues. Statistical analyses compare expression levels between patients with good prognosis (long survival) and poor prognosis (short survival), as defined by median survival time (****P < 0.0001, ***P < 0.001, **P < 0.01). **(F)** Kaplan-Meier survival analysis stratified by CKAP4 expression in HCC patients (*P* = 0.007). **(G)** Kaplan-Meier analysis of overall survival in HCC patients stratified by high and low PLOD2 expression, based on the validation cohort (*P* < 0.001). **(H)** Kaplan-Meier analysis of overall survival in HCC patients stratified by the combined CKAP4 and PLOD2 expression: low expression of both proteins, low expression of one protein (CKAP4 low + PLOD2 high or CKAP4 high + PLOD2 low), or high expression of both proteins (*P* = 0.002). All *P*-values for survival analyses were calculated using the log-rank test.

Immunohistochemistry (IHC) confirmed elevated CKAP4 and PLOD2 expression in HCC tissues compared to peritumoural tissues (Figure 4A,B,D). Higher expression of both proteins correlated with shorter survival times (Figure 4C,E), consistent with mass spectrometry results (Supplementary Figure S5A–D). Kaplan-Meier analysis revealed worse prognosis in patients with high CKAP4 and PLOD2 expression (Figure 4F,G). Additionally, CKAP4 and PLOD2 expression levels showed a positive correlation (R = 0.56, *P* < 0.001) (Supplementary Figure S4B). To further investigate their combined effect, patients in the validation cohort (N = 48) were stratified into three groups based on CKAP4 and PLOD2 expression. Group 1 (low CKAP4, low PLOD2) demonstrated the longest OS, while Group 3 (high CKAP4, high PLOD2) had the shortest OS. Group 2 (discordant expression, i.e., high CKAP4+low PLOD2 or low CKAP4+high PLOD2) showed intermediate OS (Figure 4H).

### 3.5 Establishment and evaluation of nomogram

**FIGURE 5:**
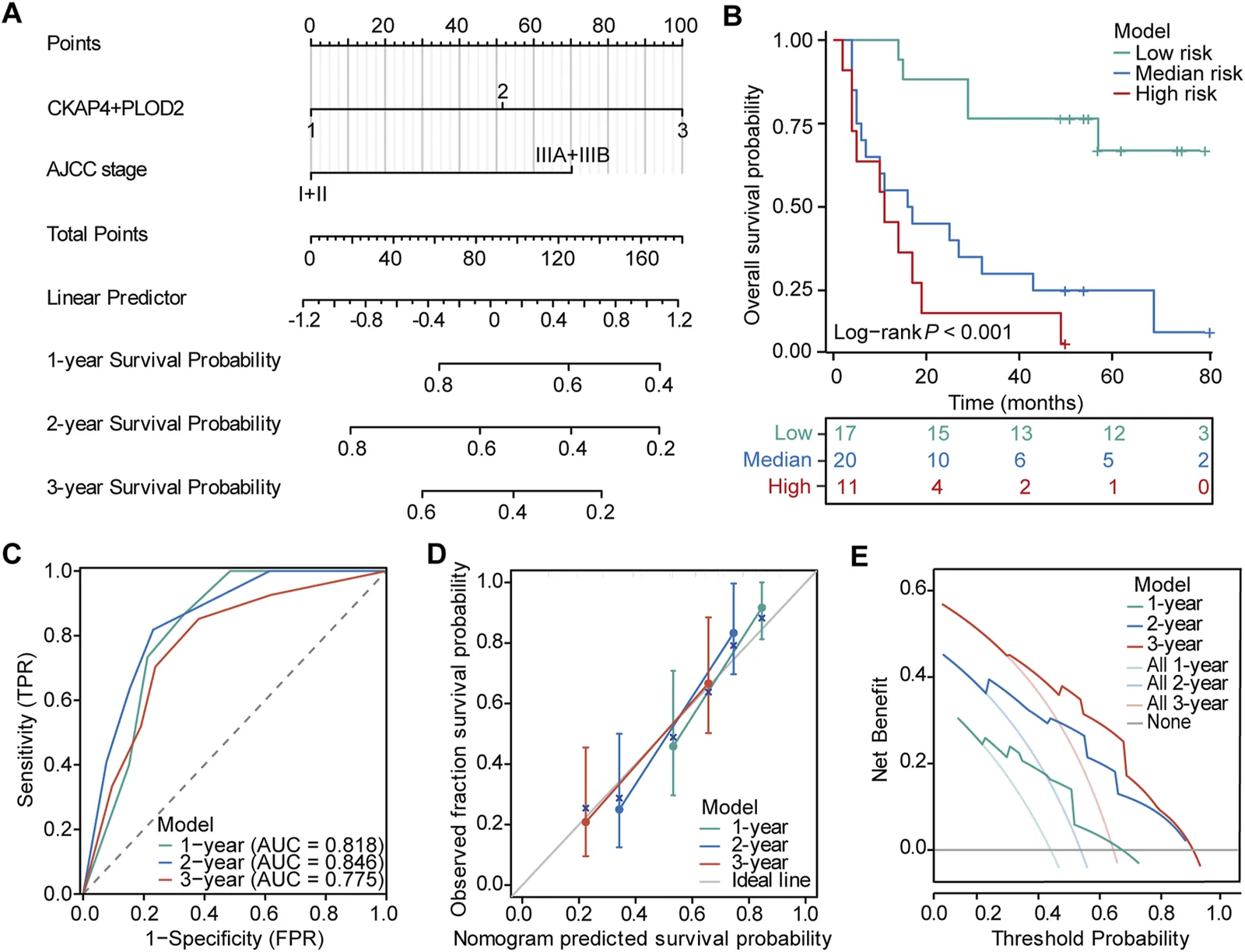
Nomogram for predicting survival probability in HCC patients. **(A)** Prognostic nomogram integrating CKAP4+PLOD2 expression with AJCC stage for predicting overall survival in hepatocellular carcinoma (HCC) patients. **(B)** Kaplan-Meier survival analysis stratified by nomogram-predicted risk groups to assess overall survival in HCC patients. **(C)** Time-dependent receiver operating characteristic (ROC) curves evaluating the predictive efficiency of the nomogram. **(D)** Calibration curves demonstrating the agreement between nomogram-predicted and observed 1-, 2-, and 3-year overall survival (OS) probabilities. **(E)** Decision curve analysis (DCA) illustrating the clinical utility of the nomogram for predicting 1-, 2-, and 3-year OS in HCC patients.

Univariate analysis identified AJCC stage, BCLC stage, CKAP4, PLOD2, and the CKAP4+PLOD2 combination as significantly associated with OS (Table 1). However, multivariate Cox regression analysis integrating these variables revealed AJCC stage (HR = 3.12, 95% CI 1.89-5.14; *P* < 0.001) and the CKAP4+PLOD2 combination (HR = 2.67, 95% CI 1.75-4.08; *P* < 0.001) as independent prognostic factors (Table 1), whereas BCLC stage lost significance (HR = 1.395, 95% CI 0.63-3.09; *P* = 0.411). These validated predictors were used to construct the primary nomogram (Figure 5A).

**TABLE 1:** Univariable and multivariable analyses of factors associated with survival in the IHC validation cohort (n = 48).

Characteristics	Univariable analysis		Multivariate analysis	
	HR (95% CI)	*P* value	HR (95% CI)	*P* value
Gender	1.237 (0.375–4.082)	0.727		
Male				
Female				
Age (years)	0.573 (0.277–1.184)	0.133		
≤55				
>55				
Edmondson-Steiner grade	1.115 (0.540–2.302)	0.769		
I + II				
III				
Tumour number	1.150 (0.441–3.001)	0.775		
Single				
Multiple				
Tumour size (cm)	2.068 (0.958–4.463)	0.064		
≤5				
>5				
AJCC stage^a^	3.000 (1.412–6.376)	**0.004**	2.529 (1.156–5.535)	**0.020**
I + II				
IIIA + IIIB				
CKAP4	2.613 (1.246–5.480)	**0.011**		
Low				
High				
PLOD2	4.118 (1.911–8.875)	**<0.001**		
Low				
High				
CKAP4+PLOD2				
All low				
Low + high or high + low	1.892 (0.684–5.232)	0.219	1.976 (0.712–5.487)	0.191
All high	4.450 (1.807–10.955)	**0.001**	3.745 (1.503–9.331)	**0.005**

^a^ American Joint Committee on Cancer 8th edition staging for hepatocellular carcinoma.

Boldface type denotes statistically significant P-values (P < 0.05).

To directly compare staging systems, we developed parallel nomograms using identical variables (AJCC/BCLC stage + CKAP4/PLOD2 combination). The BCLC-integrated model demonstrated moderate discriminatory power (1-/2-/3-year AUCs: 0.715/0.812/0.732; Supplementary Figures S6A–F), significantly underperforming the AJCC-based nomogram across all endpoints (AUCs: 0.818/0.846/0.775; Figure 5C). This systematic comparison (visualized in Figure 5C vs. Supplementary Figure S6B) confirmed AJCC’s superior integration with molecular markers. Furthermore, ROC analysis demonstrated the integrated nomogram’s enhanced predictive capacity over individual indicators (Supplementary Figures S7A–C), validating its clinical utility for HCC risk stratification. The C-index for the OS nomogram was 0.738 (95% confidence interval [CI], 0.698–0.779). Calibration curves demonstrated good agreement between predicted and actual 1-, 2-, and 3-year OS probabilities in the validation cohort (Figure 5D). DCA showed that the model could improve the net benefit and exhibit a broader threshold probability in predicting the prognosis of HCC patients (Figure 5E).

Based on the established nomogram, we calculated the total score for each patient in the validation set and used the best cutoff points of the total score to establish risk stratification. Patients were categorized into high-risk (>121.85), medium-risk (>51.59 and ≤121.85), and low-risk (≤51.59) groups. Survival analysis showed significantly better prognosis in the low-risk group compared to the high-risk group (*P* < 0.001, Figure 5B).

## 4 Discussion

HCC remains a significant global health challenge, characterized by high incidence and poor prognosis despite considerable advances in treatment modalities over recent decades. The paucity of robust prognostic markers underscores the pressing need for a distinct and reliable predictive model. In this study, we leveraged mass spectrometry-based proteomics to unveil the molecular heterogeneity of HCC and identified a significant association between the expression of CKAP4 and PLOD2 and OS. Furthermore, our analysis revealed that AJCC stage may also serve as a potential prognostic factor, aligning with previous findings in HCC research (Ouyang et al., 2020; El‐Fattah et al., 2016).

In the development of prognostic models for HCC, serum biomarkers such as AFP and protein induced by PIVKA-II are commonly prioritized for inclusion. While classically prognostic in HCC, AFP lost independent significance when combined with tissue proteomics, consistent with evidence suggesting serum biomarkers’ limited value in tissue-enriched cohorts, particularly early-stage HCC. This may reflect biological redundancy or sample size limitations. PIVKA-II was excluded due to unavailable retrospective data. Future studies will prospectively integrate serum and tissue biomarkers to enhance prognostic precision.

We developed a personalized prognostic nomogram and established risk stratification criteria to aid in the clinical management of HCC patients at initial diagnosis. Our comprehensive analytical assessment confirmed the model’s excellent ability to differentiate and calibrate, effectively predicting OS and accurately stratifying HCC patients by risk in our dataset. This tool has the potential to assist clinicians in making informed decisions regarding treatment strategies and follow-up protocols.

Our findings indicate that CKAP4 and PLOD2 are critical prognostic molecules in HCC, with significant overexpression associated with poor prognosis. CKAP4, also known as p63, is localized in the endoplasmic reticulum and plays a crucial role in maintaining its structure (Shibata et al., 2010). The protein is categorized into two main subtypes: TAp63 and ΔNp63. While TAp63 belongs to the p53 family and exhibits tumour-suppressive functions, the ΔNp63 subtype is often upregulated in tumour cells and associated with poor prognosis and disease progression (Wu et al., 2003; Carroll et al., 2006). This upregulation has been reported in various cancers, including pancreatic ductal adenocarcinoma, lung adenocarcinoma, and squamous cell carcinomas of the lung and esophagus (Kimura et al., 2016; Shinno et al., 2018). PLOD2, a member of the PLOD family responsible for lysine hydroxylation, is localized in the rough endoplasmic reticulum and involved in collagen post-translational modification. Recent studies have implicated the PLOD family, particularly PLOD2, in tumour progression, invasion, and metastasis (Wang et al., 2021; Jiang et al., 2020; Eisinger-Mathason et al., 2013). Elevated expression of both PLOD1 and PLOD2 has been associated with poor prognosis and increased invasiveness in HCC (Yang et al., 2023; Li et al., 2023). PLOD2, as a key enzyme promoting stable collagen cross-linking, plays a crucial role in facilitating tumour cell motility, invasion, and proliferation (Du et al., 2017; Kiyozumi et al., 2018; Yang et al., 2020; Qi and Xu, 2018).

Our study revealed a positive correlation between CKAP4 and PLOD2 expression in HCC, a finding that has not been previously reported. However, the interactions between these proteins in HCC and their exact molecular mechanisms in tumour regulation require further investigation. IHC results elucidated the expression patterns of CKAP4 and PLOD2 in HCC tumour tissues and surrounding areas, providing visual evidence of their upregulation. Cox regression analysis confirmed that the combination of CKAP4 and PLOD2 is an independent prognostic factor for OS in HCC patients, suggesting that overexpression of these proteins may predict shorter survival time and poorer prognosis.

This molecular prognostic signature synergizes with clinical staging systems to enhance predictive precision. Multivariate analysis confirmed that the AJCC staging system has superior prognostic independence compared to BCLC, which may be attributed to its detailed classification of tumour invasiveness and metastasis. Model comparisons further validated the enhanced predictive accuracy of the AJCC-based nomogram, indicating a stronger alignment with the biological characteristics of hepatocellular carcinoma progression. The nomogram developed in this study incorporates AJCC stage, a recognized risk factor in many HCC prediction models (Wang et al., 2023), with CKAP4 and PLOD2 expression levels. While current tissue-based approaches (including our DIA proteomics) may not fully resolve intratumoral heterogeneity compared to advanced spatial techniques like laser-capture microdissection, this model overcomes AFP’s critical limitation in capturing tumour microenvironment architecture. The integration of AJCC staging with CKAP4/PLOD2 spatial expression patterns achieved superior prognostic accuracy compared to serum biomarkers, with the combined model demonstrating greater predictive power than individual factors in assessing HCC prognosis.

The model’s postoperative prognostic stratification capability should be interpreted within current therapeutic paradigms. Although our model primarily emphasizes postoperative prognosis stratification, the selection of treatment modalities remains a critical consideration, especially for advanced hepatocellular carcinoma (BCLC stage C). These molecular insights must be contextualized within evolving clinical paradigms. Despite current guidelines recommending systemic therapy (such as tyrosine kinase inhibitors and immune checkpoint inhibitors) for patients with BCLC-C, emerging evidence suggests that surgical resection may be beneficial for specific cases with vascular invasion but no extrahepatic spread (Govalan et al., 2021; Famularo et al., 2022; Zhang et al., 2019). Remarkably, 84% (26/31) of our discovery cohort received adjuvant therapy post-resection—primarily TACE (16 cases) and radiotherapy (4 cases)—reflecting prevailing Asian HCC management patterns that favor aggressive local control even in advanced stages (Zhang et al., 2019).

In this complex landscape, nomograms serve as visual tools for personalized risk assessment in oncology, addressing the complexity of balancing various clinical variables while avoiding individual physician bias (Balachandran et al., 2015). They are particularly valuable when the benefits of additional treatments are uncertain and can aid in individualized risk stratification and clinical decision-making (Weiser et al., 2008; Rudloff et al., 2010). In the context of HCC, where treatment decisions can be complex and multifaceted, such tools provide new ideas and strategic insights for prognostic prediction.

Our study’s strengths include the use of DIA mass spectrometry-based proteomic analysis, validation in an independent cohort, rigorous statistical analysis including Cox and LASSO regression, and the development of a prediction model with good performance, fewer variables, and clinical accessibility. This approach not only prevents model overfitting but also improves its clinical applicability and accuracy (Zhu et al., 2020). However, limitations include a small sample size and the absence of external validation for the nomogram. Notably, although we stratified HCC patients into early-intermediate (BCLC-A/B; AJCC I/II) and advanced stages (BCLC-C; AJCC III), the limited subgroup sizes precluded robust stage-specific analyses. To address these constraints, we are prospectively implementing a multicenter validation study with expanded cohorts to enable statistically powered stratification across disease stages.

## 5 Conclusion

In conclusion, this study suggests that AJCC stage and expression levels of CKAP4 and PLOD2 significantly impact HCC prognosis. The developed nomogram demonstrates effective OS prediction and risk stratification in our dataset, potentially guiding tailored treatment and surveillance strategies. The application of this liver cancer risk prediction model is of great significance in individualizing treatment and regular testing for HCC patients.

## Data Availability

The datasets presented in this study can be found in online repositories. The names of the repository/repositories and accession number(s) can be found in the article/Supplementary Material.
