# Supplementary material for: CKAP4 and PLOD2 as novel prognostic biomarkers in hepatocellular carcinoma: a proteomics-driven risk stratification model
Source: Front Cell Dev Biol. 2025 Jul 2;13:1577161. doi: 10.3389/fcell.2025.1577161 (PMC12264339; doi:10.3389/fcell.2025.1577161)
Supplement: Supplementary file 3 [file Supplementaryfile1.docx]

Supplementary Material

# Supplementary Figures and Tables

## Supplementary Figures


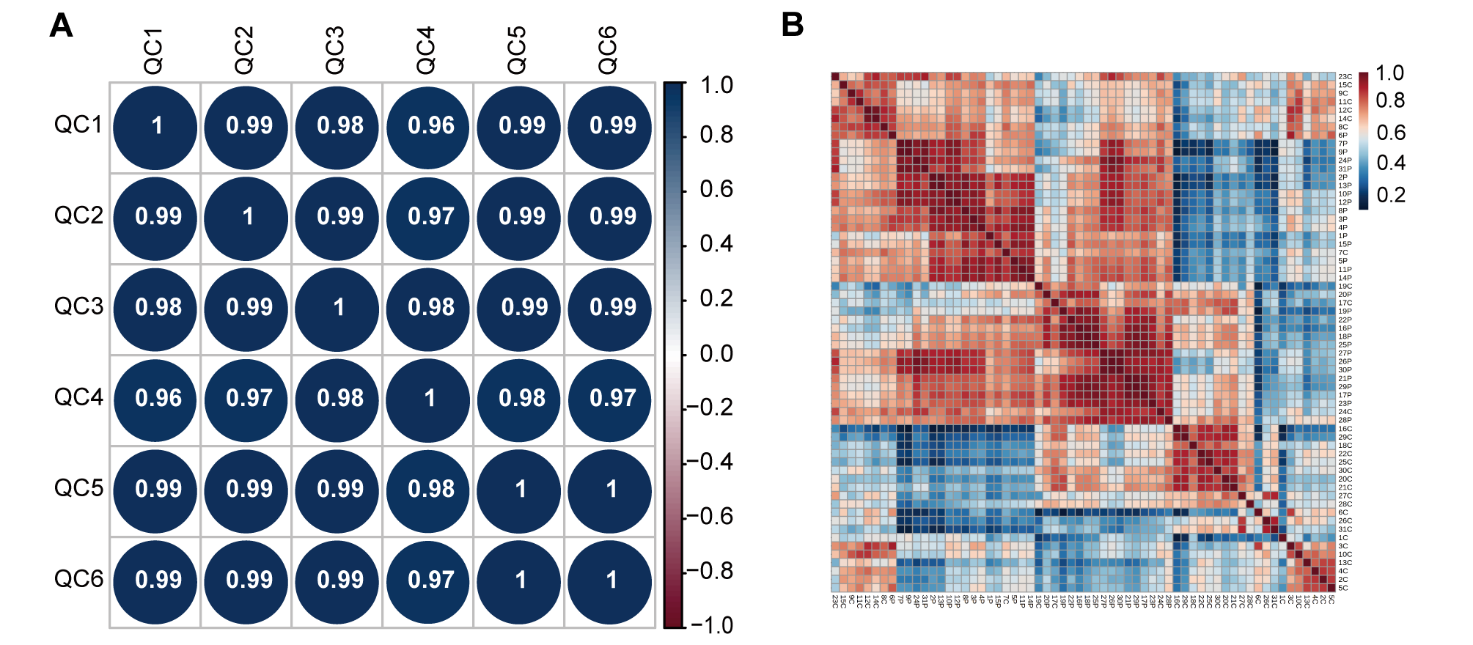


**Supplementary Figure 1. Quality Control Analysis of Proteomic Samples. (A)** Scatter plot matrix of Pearson correlation coefficients for protein quantities in the proteomics dataset. Blue dots indicate positive correlations, while red dots represent negative correlations. **(B)** Heatmap visualization of Pearson correlation coefficients among protein samples, demonstrating the overall similarity and reproducibility of the proteomic profiles. C: tumors; P: adjacent normal tissues.


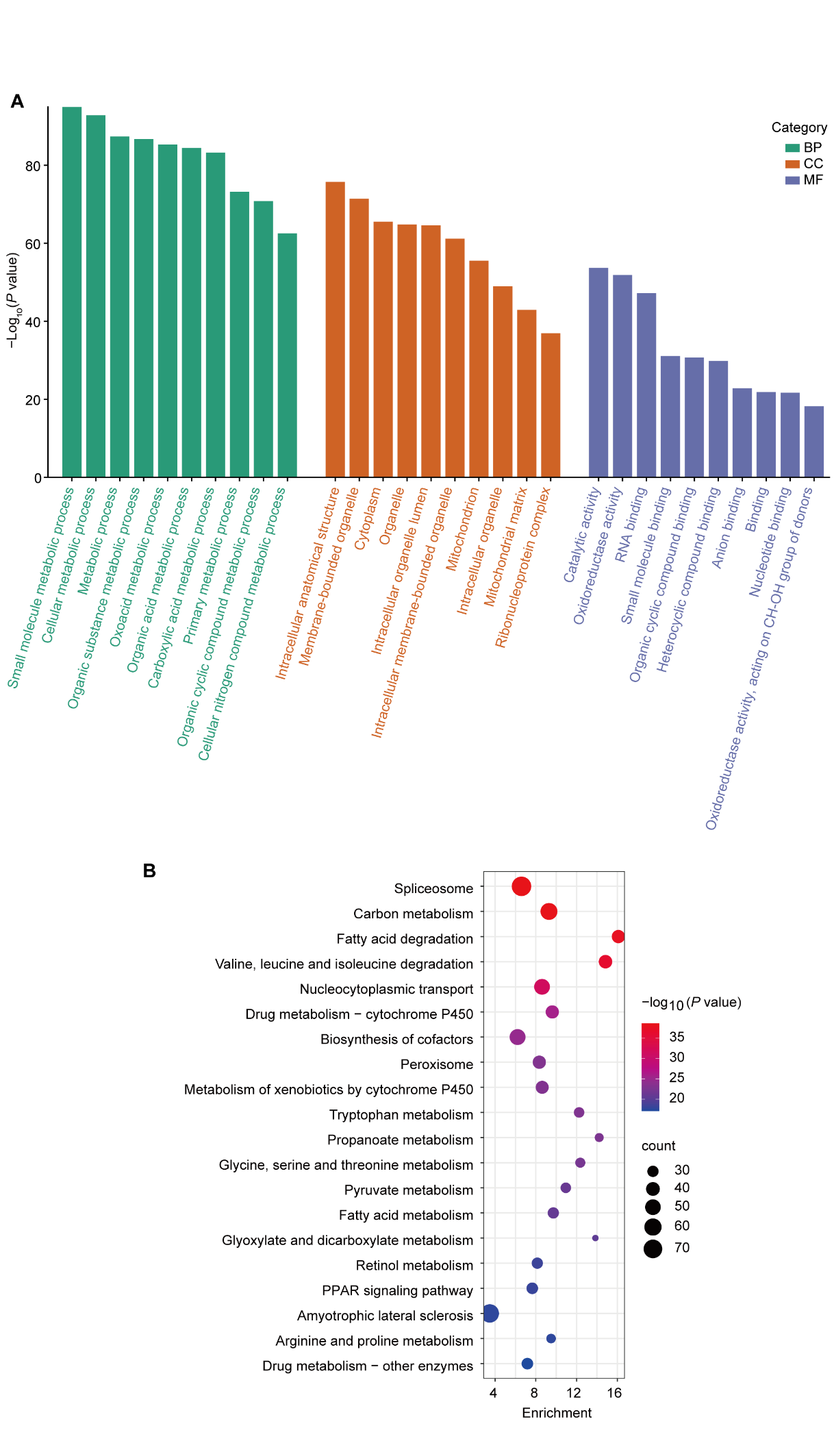


**Supplementary Figure 2. Functional enrichment analysis of common differentially expressed proteins (DEPs). (A)** Bar plot showing the top 10 significantly enriched Gene Ontology (GO) biological processes associated with the identified DEPs. The x-axis represents the -log10(*p*-value), and the y-axis lists the enriched biological processes. **(B)** Bubble plot depicting the top 20 significantly enriched Kyoto Encyclopedia of Genes and Genomes (KEGG) pathways for the DEPs. The size of each bubble represents the number of DEPs involved in the pathway, while the color intensity indicates the significance level (-log10(*p*-value)).


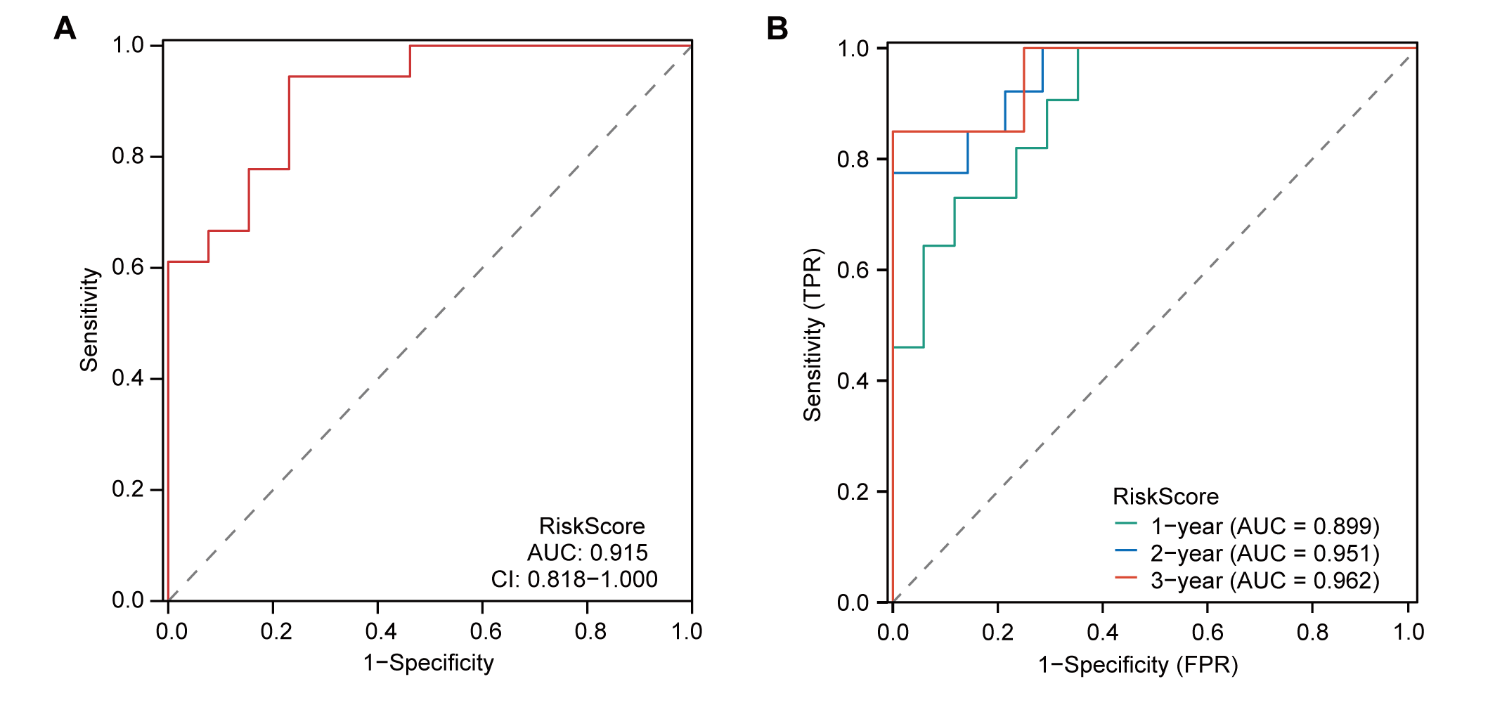
**Supplementary Figure 3.** **Development and evaluation of the prognostic model. (A)** Receiver operating characteristic (ROC) curve analysis of the LASSO-Cox regression model for predicting overall survival (OS). The area under the curve (AUC) is presented to quantify the model's discriminative ability. **(B)** Time-dependent ROC curve analysis of the LASSO-Cox regression model for predicting overall survival at multiple time points. The AUC values for 1-year, 2-year, and 3-year OS predictions are shown separately, demonstrating the model's performance over different time horizons.


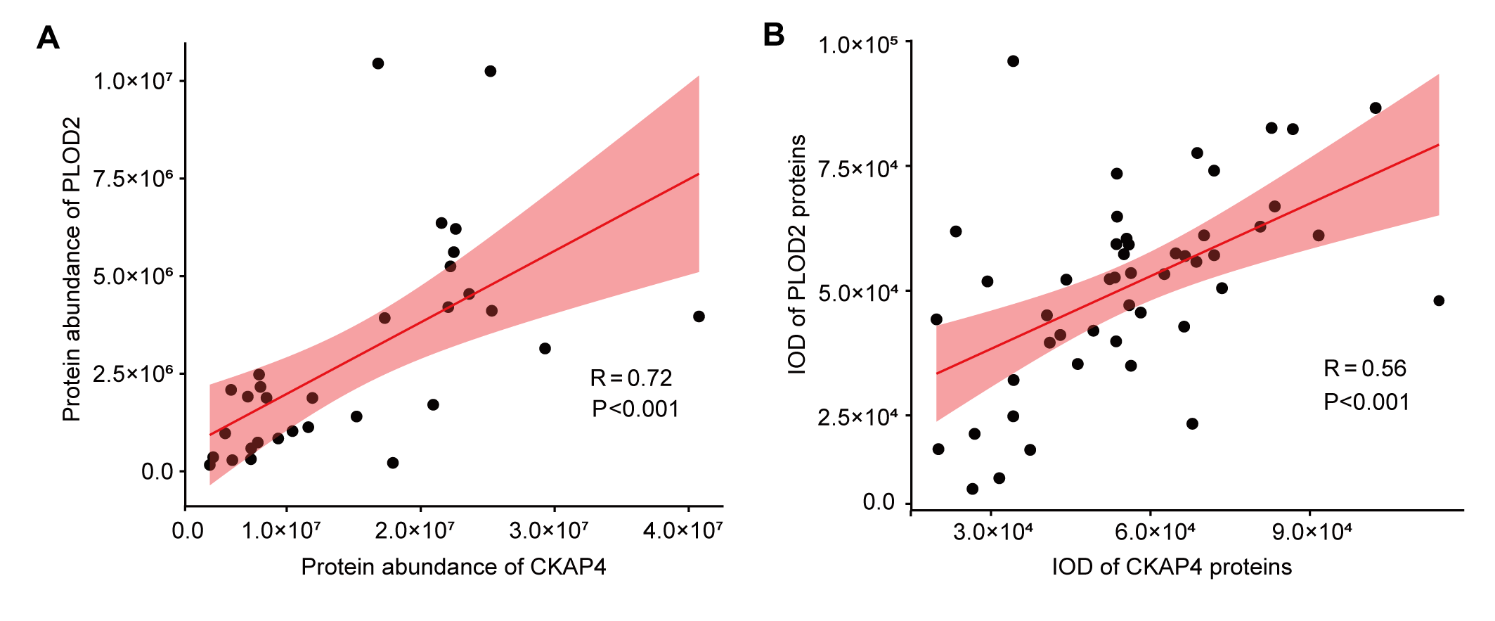


**Supplementary Figure 4. Correlation between PLOD2 and CKAP4 expression in liver tissues. (A)** PLOD2 and CKAP4 expression levels in liver tissue samples were quantified using data-independent acquisition (DIA) mass spectrometry. **(B)** Scatter plot illustrates the positive correlation between CKAP4, and PLOD2 protein expression levels as determined by immunohistochemistry. The Spearman's rank correlation coefficient (r) is indicated.

**
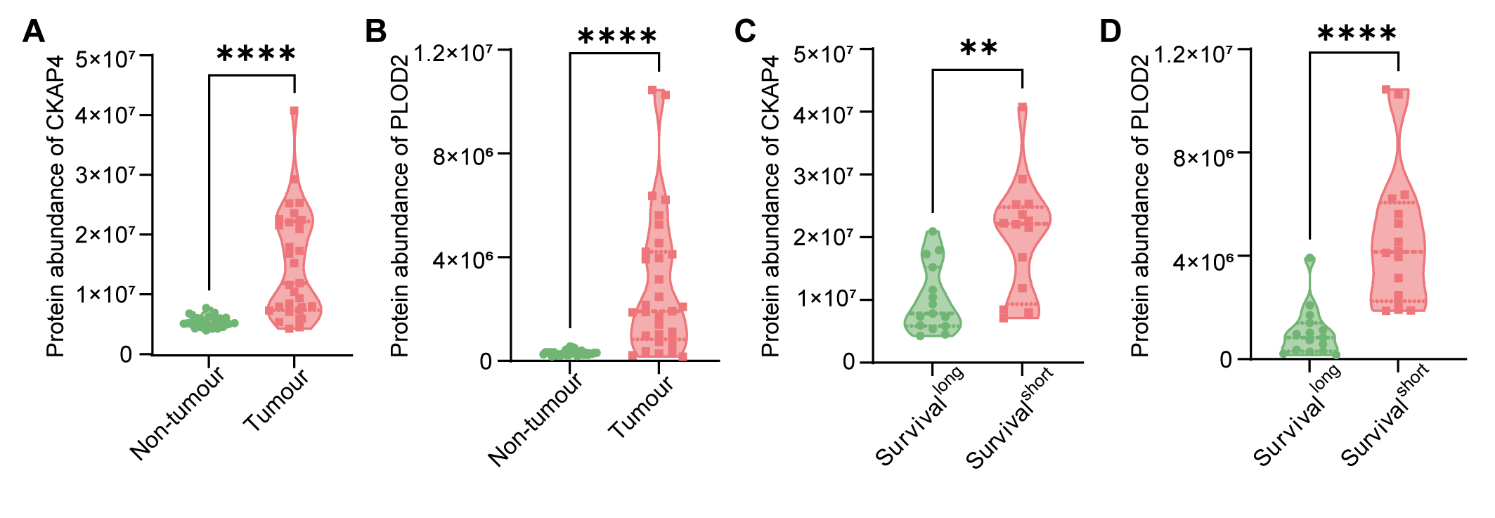
**

**Supplementary Figure 5. Association of CKAP4 and PLOD2 protein abundance with overall survival in hepatocellular carcinoma (HCC). (A-B)** Differential expression of CKAP4 (A) and PLOD2 (B) in paired peritumoural and HCC tissues (*****P* < 0.0001). **(C-D)** Comparison of CKAP4 (C) and PLOD2 (D) expression levels between patients with favorable (long survival) and poor (short survival) prognosis, as determined by mass spectrometry. Grouping was based on median survival time (***P* < 0.01, *****P* < 0.0001).


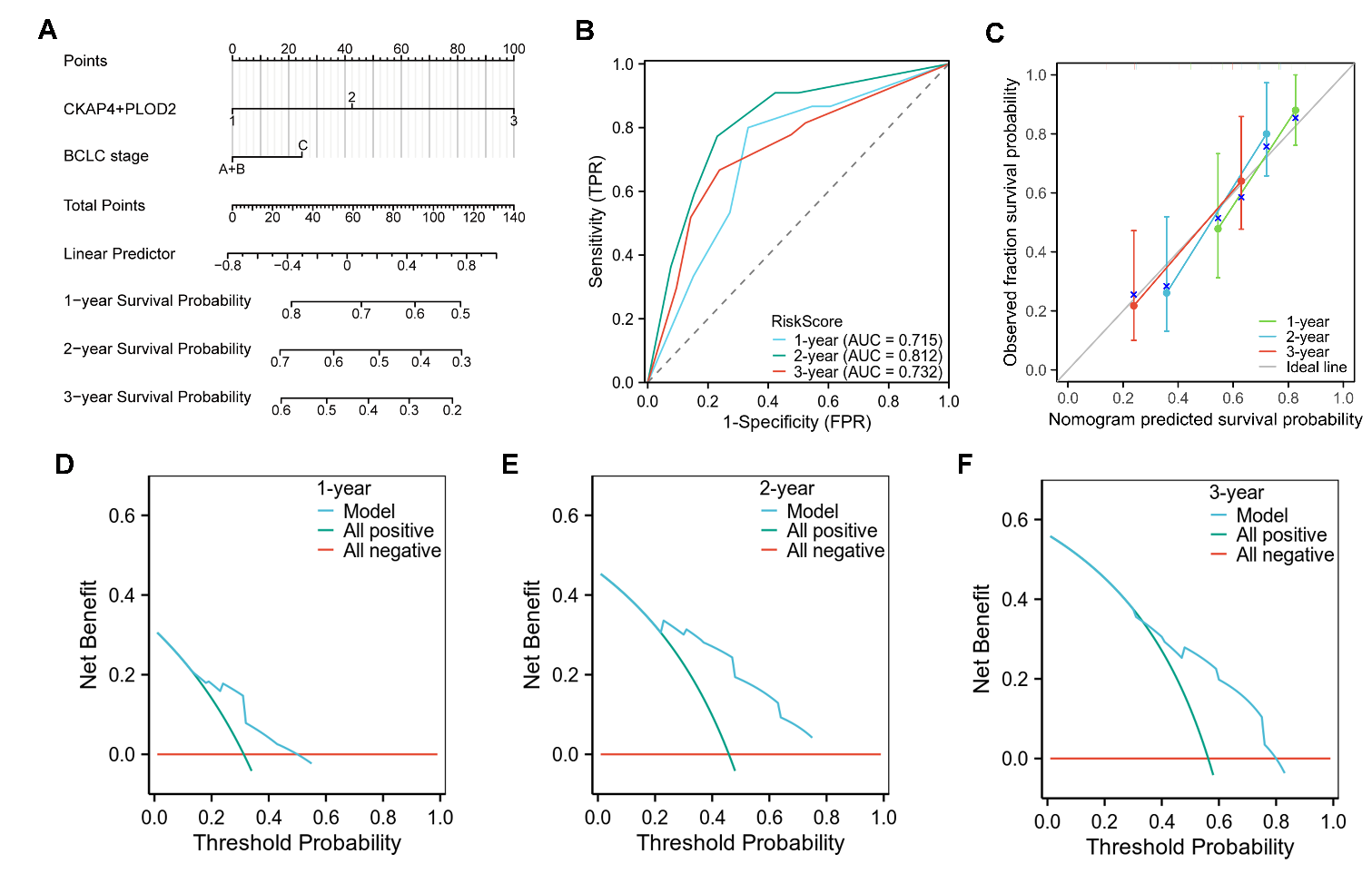


**Supplementary Figure 6. Nomogram integrating BCLC stage and 'CKAP4+PLOD2 biomarker' combination for predicting postoperative survival in hepatocellular carcinoma (HCC) patients.** (A) Nomogram of predicting the OS of patients with hepatocellular carcinoma by combining CKAP4+PLOD2 expression with BCLC stage. (B) The receiver operating characteristic (ROC) curves at 1-, 2- and 3 years were used to evaluate the predictive efficiency of nomogram. (C) Calibration curves showing the differences between the 1-, 2-and 3-year overall survival probabilities predicted by the nomogram and those observed. (D-F) Decision curve analysis (DCA) was used to analyze the clinical application of nomogram in predicting 1-, 2- and 3-year OS in HCC patients.


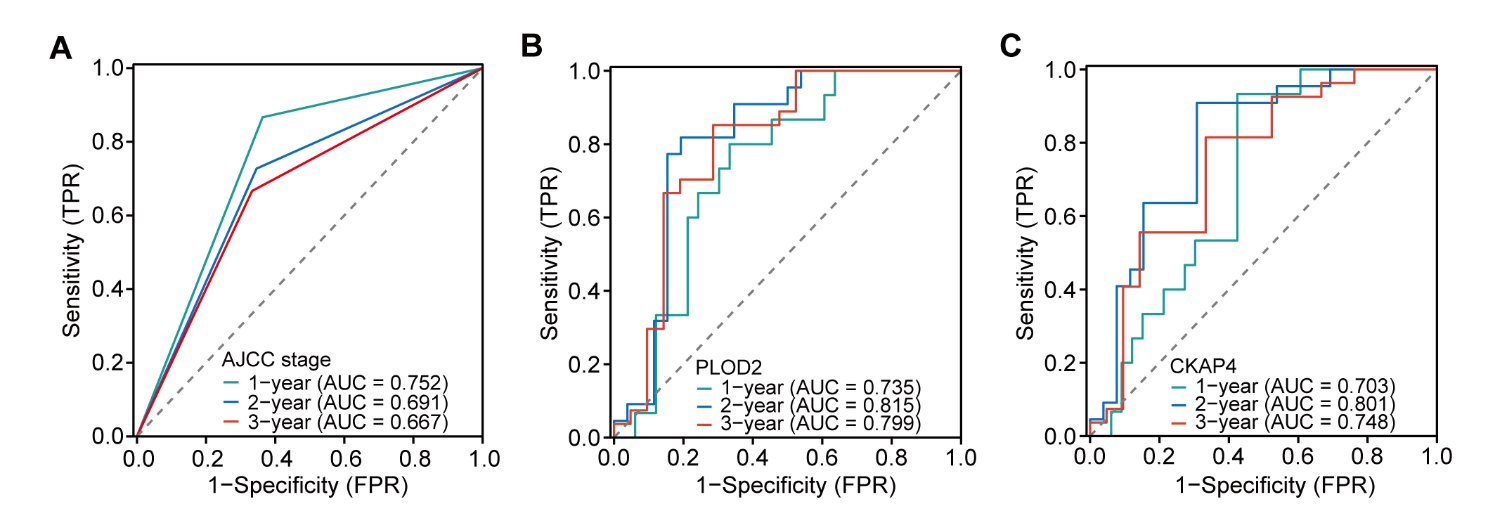


**Supplementary Figure 7. Time-dependent receiver operating characteristic (ROC) curves for predicting hepatocellular carcinoma (HCC) progression. (A-C)** ROC curves illustrating the predictive performance of (A) AJCC stage, (B) PLOD2 expression, and (C) CKAP4 expression for HCC progression.

## Supplementary Tables

**Supplementary Table 1.** **Association between CKAP4 and PLOD2 expression and clinicopathological features in the DIA discovery cohort (n=31).**

**Supplementary Table 2. Association between CKAP4 and PLOD2 expression and clinicopathological features in the IHC validation cohort (n=48).**

**Supplementary Table 3. Differentially expressed proteins in hepatocellular carcinoma.**

**Supplementary Table 4. Candidate prognostic biomarkers identified from differentially expressed proteins.**

**Supplementary Table 5. Prognostic-related genes identified from The Cancer Genome Atlas (TCGA) database.**

**Supplementary Table 6. Common prognostic factors identified across multiple analyses.**
